# Fava bean and buckwheat are sustainable food sources which support satiety and beneficially modulate several biomarkers, bacteria and metabolites associated with human health

**DOI:** 10.1007/s00394-025-03726-6

**Published:** 2025-06-07

**Authors:** Madalina Neacsu, Marietta Sayegh, Nicholas J. Vaughan, Gary J. Duncan, Louise Cantlay, Susan Anderson, Donna Henderson, Claire Fyfe, Freda Farquharson, Salifu Saibu, Graham Horgan, Petra Louis, Alexandra M. Johnstone, Wendy R. Russell

**Affiliations:** 1 The Rowett Institute, University of Aberdeen, Aberdeen, AB25 2ZD UK; 2 Biomathematics and Statistics Scotland, AB25 2ZD, Aberdeen, UK

**Keywords:** Coprococcus eutactus, Salicylic acid, 2,3-dihydroxybenzoic, Homocysteine, Nutrient diversification, Fasting glucose & insulin

## Abstract

**Background:**

The world’s population requires adequate food supply, satisfying specific nutrient requirements to meet dietary recommendations, promote nutrition security and sustain health, while stimulating agriculture biodiversity. This study assessed the potential of buckwheat and fava bean to diversify the source of dietary nutrients.

**Methods:**

Twenty healthy volunteers (*n* = 6 men, *n* = 14 women; 42.08 ± 12.12 years; body mass index 24.72 ± 4.69 kg/m^2^) were recruited in a randomised controlled crossover study consisting of two seven-day intervention periods, buckwheat- and fava bean-based diets were provided to meet individual volunteers resting metabolic rate requirements. The study assessed subjective hunger and the impact of the diets on the gut microbiota composition and the plasma profiles of lipids, glucose, insulin, urea and homocysteine. Plasma, urine and faecal metabolites were also measured before and after consumption of each diet using targeted metabolomics (LC- and GC-MS).

**Results:**

Both intervention diets were as satiating as the volunteers’ habitual diets (*p* = 0.234). The fava bean diet significantly reduced fasted plasma glucose and insulin and increased plasma homocysteine (*p* < 0.05). Buckwheat diet decreased plasma homocysteine (*p* < 0.01) and increased plasma, urine and faecal concentrations of salicylic acid and 2,3-dihydroxybenzoic acid. Both diets significantly increased plasma non-esterified fatty acids values, reduced plasma urea and faecal deoxycholic acid concentrations (*p* < 0.05). The fava bean diet provided significantly higher amounts of dietary fibre (both in comparison with habitual and buckwheat diet) significantly increasing the urine indole-3-propionic acid concentration (*p* < 0.01) (Day 0 vs. Day 7) and the faecal, plasma and urine indole-3-propionic acid concentrations (*p* < 0.01) (on Day 7 buckwheat vs. Day 7 fava bean diet). Furthermore, the fava bean diet promoted the growth of the gut bacterium *Coprococcus eutactus* (*p* < 0.05).

**Conclusion:**

Buckwheat and fava bean contribute in a sustainable way to meet dietary recommendations and to promote dietary diversification. Diets rich in buckwheat and fava bean were found to be satiating and to beneficially modulate several biomarkers, bacteria and metabolites which are correlated with prevention of metabolic disorders such as cardiovascular disease and type 2 diabetes.

**Supplementary Information:**

The online version contains supplementary material available at 10.1007/s00394-025-03726-6.

## Introduction

With a growing global population and climate change challenges, providing an adequate food supply and nutritional security is a top global priority. The availability of sufficient nutrients (i.e. dietary protein) of adequate quality is undeniably a source of concern for human health [1]. A promising way to achieve nutritional security is to explore sustainable and diverse food supplies in response to the predicted global food crisis and other environmental and economic issues [1, 2].

Alongside the impact of diet on planetary health, inadequate nutrition poses a risk factor for metabolic disorders such as type 2 diabetes (T2D), cardiovascular disease (CVD), and death, as these have been related to higher consumption of low-quality of nutrients [3–6]. Diversifying nutrient sources by increasing the consumption of plant-based foods has been associated with lowered blood pressure and low-density lipoprotein (LDL), enhanced insulin sensitivity, decreased rates of obesity, and a lower risk of death from all causes [7, 8]. The world’s population requires food sources which are diverse, sustainable, and nutritious. Therefore, plant-based sources could contribute to addressing the dietary needs of a growing global population, if they can satisfy hunger and contribute to meeting nutritional needs [9].

Plant-based foods are essential for the delivery of dietary fibre, recognised for contributing to lowering the risk of developing CVD and T2D [10, 11]. Plant-based foods are also abundant in bioactive components including phytosterols, phytoestrogens, flavonoids, carotenoids and other phytophenols considered to beneficially impact on chronic disease development [12–14]. These bioactives, along with plant macronutrients and their metabolites are well researched in pre-clinical and human studies with several reviews summarising the key aspects related to CVD and T2D [15–18]. Existing research already highlights a key role of amino acid metabolism, as well as non-nutrient phytochemicals in the early pathogenesis of T2D and CVD and suggest that they could aid in risk assessment [19]. Microbial-derived metabolites of aromatic amino acids found in plasma such as indole-3-propionic acid was also associated with a lower likelihood of developing T2D [20] in people with impaired glucose tolerance. Furthermore, a diet rich in fibre contributes to maintaining a healthy gut microbiota associated with increased diversity and the production of short chain fatty acids (SCFA) in the colon, mainly acetate, propionate and butyrate [21]. Butyrate is a primary energy source for the enterocytes and modulates immune activity, while acetate and propionate mainly exert systemic regulatory functions [21]. Microbial SCFA exert several beneficial effects on human metabolism by intervening in glucose homeostasis, lipid metabolism and appetite regulation [22].

Certain crops such as buckwheat, hemp, fava bean, lupin and peas are good sources of dietary protein, fibre and micronutrients [23, 24].

Buckwheat (*Fagopyrum esculentum*) has been extensively studied for its functional and characteristic properties as a food ingredient and has huge potential for use in the functional and clinical food industries [24–26]. Buckwheat is a quality source of protein, dietary fibre, resistant starch, fagopyritols, minerals, and vitamins [25, 26]. Buckwheat is also known to contain well-balanced amounts of essential amino acids such as methionine, tryptophan and lysine [26]. It is also reported that buckwheat consumption in humans is linked to a lower prevalence of hyperglycaemia and increased glucose tolerance in individuals diagnosed with diabetes [27]. Furthermore, the consumption of buckwheat-rich meals (bread) resulted in a significant reduction of hunger postprandially in healthy volunteers [9].

Fava bean (*Vicia faba*) is an adaptable crop and can be grown in various climatic regions [28]. Fava bean is high in protein (around 22%) and a good source of dietary fibre (approx. 10% as total NSP). Fava bean is also a good source of bioactive phytochemicals including kaempferol, quercetin, myricetin, tyrosol, catechin, epicatechin and cyanidin, which have been found to be potentially beneficial to human health [23, 29]. Legumes like fava bean, can be combined with other plant-based foods to diversify the dietary protein [30] since they include several essential amino acids [19]. The consumption of high protein meals (bread) prepared with fava bean flour resulted in similar hunger scores as meat-based meals when consumed by healthy volunteers [9]. Therefore, even though, there is promising (mainly indirect) evidence that fava bean rich diet consumption could support satiety and gut health, there is still a gap in fava-specific research, especially in humans, therefore this would benefit from well-designed nutritional intervention studies. Similarly, while buckwheat likely has beneficial effects on hunger regulation and gut microbiota due to its fibre, resistant starch, and polyphenols, the scientific evidence is still sparse, especially in human-specific intervention studies, running buckwheat-rich focused dietary feeding trials.

This study assessed the potential of two sustainable sources of nutrients, buckwheat and fava bean to diversify and contribute towards meeting dietary needs, as well as subjective hunger. The study also measured the concentrations of the fasted plasma profiles of lipid, glucose, insulin, urea and homocysteine following the consumption of seven-day buckwheat and fava bean rich diets by healthy volunteers together with additional plasma, urine and faecal metabolites measured before and after consumption of each diet. Furthermore, the study assessed the impact of diets on the composition of the volunteers’ gut microbiota.

## Subjects and methods

### Subjects

Twenty 18–65 years old healthy male and female volunteers with a body mass index (BMI) ranging from 18 to 35 kg/m^2^ were recruited to participate in a randomised crossover intervention study. The exclusion criteria included volunteers who smoke, consume an exclusively vegetarian diet, with food allergies, or take medication, including antibiotics (three months prior the intervention). Individuals with certain medical conditions such as diabetes, gastrointestinal disorders, kidney disease, hepatic disease, favism, alcohol or substance abuse were also excluded from the study.

**Fig. 1:**
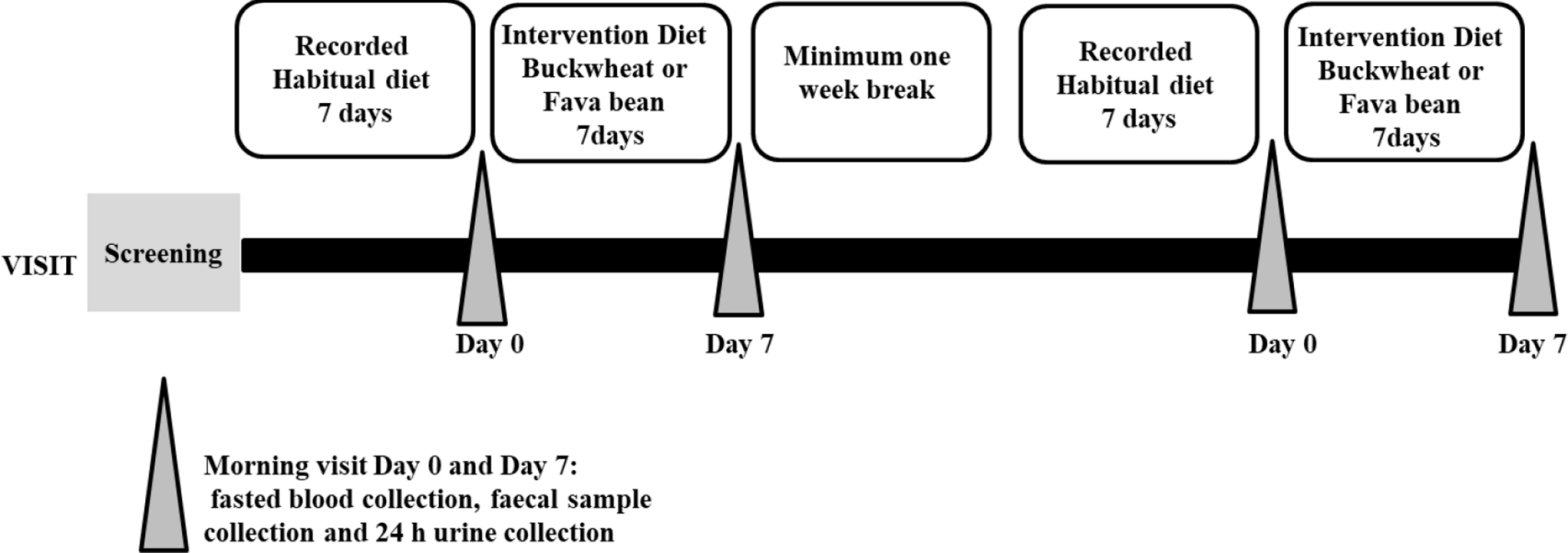
Human dietary intervention study diagram. Each volunteer (*n* = 20) was randomised to consume two one-week intervention diets. For each diet, all the meat was replaced for one week either with buckwheat or fava bean. One week before each intervention diet, participants recorded a weighed dietary intake of their habitual diet. On the morning of the first day of each intervention diet and following the seventh day, the volunteers provided a faecal sample, an overnight fasted blood sample, and a 24-hour urine collection sample. The intervention diet meals consisted of a four-day rotation menu. Between the intervention diets, there was a minimum washout period of seven days

The study took place between May 2014 and October 2015. The study was approved by the Rowett Institute Ethical Committee, University of Aberdeen, U.K. After signing a consent form, the volunteers participated in five visits to the Human Nutrition Unit (HNU) at The Rowett Institute (RI), Aberdeen, U.K., from which one was the screening visit and four were morning intervention visits. As part of the screening visit, volunteers’ height was measured to the nearest 0.1 cm with the use of a stadiometer (Holtain Ltd, Crymych, Dyfed, U.K.) and their weight was measured to the nearest 100 g on a digital scale (DIGI DS-410; C.M.S. Weighing Equipment, London, UK) and all volunteers completed a self-reported medical questionnaire. Furthermore, the body composition and resting metabolic rate (RMR) were measured under standardised conditions [20]. A fasting blood sample was also collected to assess for glucose-6-phosphate dehydrogenase (G6PD) deficiency to rule out the presence of undiagnosed favism. Eligible volunteers participated in two one-week, randomised cross-over dietary intervention diets, with all food provided by the HNU, with a minimum washout period of two weeks between the diets. The participants attended the HNU at Day 0 and after Day 7 of each intervention diet to provide blood, urine and faecal samples (Fig. 1).

### Study intervention visits

Prior to each intervention the volunteers recorded their habitual diet in a seven-day weighed food diary. The volunteers were instructed to come after an overnight fast to the HNU and provided faecal, blood and 24-hour urine samples. These samples were collected before (Day 0) and after (Day 7) each intervention diet week.

Hunger and appetite were measured hourly during waking hours (0700–2300) with the use of visual analogue scales (VASs) using a 100 mm scale and the following formula: [hunger + (100 - fullness) + prospective consumption + desire to eat]/4, as previously described [31] and [32] (during both the habitual diet weeks and the intervention weeks (when the fava bean-based and buckwheat-based diets were consumed). The questionnaires included in the food diaries, in paper form, and every hour contained six questions where the volunteers were asked on motivation to eat, all in the line scale format related to hunger, fullness, desire, and quantity consumed. Scales were recorded from, for example, “not at all hungry” to “extremely hungry,” so that higher scores indicated more intense subjective sensations. These questionnaires were completed hourly by the subjects in each day of the study; subjects were instructed to allow at least five hours between breakfast and lunch and lunch and dinner and to consume all of the food provided.

The study was conducted in accordance with the Declaration of Helsinki and registered with clinicaltrial.gov (study I.D. number: NCT06406270). Volunteers were recruited between May 2014 and September 2015.

### Intervention diets

On Day 0 of each intervention diet, volunteers were provided with meals, which they consumed for seven consecutive days in a four-day rotation menu with four different daily diet menus which repeated after day four (on day one to four they received diet day one to four and on day five to seven they received diet day one to three again). Participants were instructed to consume only the meals that were provided to them during the study days. The breakfast, lunch and dinner meals of the diets were designed to contain 30% fat, 15% protein, 55% carbohydrate, at seven different energy levels: 1500, 1750, 2000, 2250, 2500, 2750 and 3000 kcal/day. The energy requirements for each volunteer were calculated based on the volunteers’ RMR measurements and ensured that the meals provided for each volunteer were at the closest match to their energy needs (receiving one of the seven energy level diets), but the same amount of buckwheat and fava bean food products were given to the volunteers regardless of their energy requirements (80 g per meal). For the intervention diets, all meat was replaced with either buckwheat or fava bean food products. The composition of each meal, in terms of energy, fat, carbohydrate, and protein, was calculated by using McCance and Widdowson’s the Composition of Foods [33]. Supplementary **Table S1** provides the menu composition of the intervention diet (2000 kcal menu). During the intervention diets all drinks such as coffee, juice and tea were restricted and provided by HNU, but the volunteers were allowed to drink water *ad libitum*. Alcoholic drinks were not allowed to be consumed during the intervention diet weeks. During the intervention diet weeks, all food and drink consumed, including any leftovers, were recorded in a provided food diary.

### Composition of intervention diets

The breakfast, lunch and dinner of each day of the four-day rotational 2000 kcal/day representative menu were combined, generating four days of buckwheat and fava bean diets respectively. The 2000 kcal day was chosen as this is often considered standard and used by food industry to display on the food products nutritional label. The four days of the 2000 kcal menus were lyophilised (Heto Lab Equipment; Allerød; Denmark) freeze milled (Spex 6700; Edison; U.S.A.), vacuum packed and stored at − 20 °C stored prior to analysis. The protein (measured as crude nitrogen), including amino acid composition (excluding tryptophan); dietary fibre (measured as soluble and insoluble non-starch polysaccharide (NSP)), total fat content, micronutrient minerals and a range of phytochemicals were measured by standard published protocols as described previously [34]. Total carbohydrate content in the diet was determined following hydrolysis and available carbohydrates are broken down to their constituent monosaccharides. The released glucose is determined by a glucose oxidase procedure [35]. The study diets were also analysed using WinDiets Nutritional Analysis Software Suite (Version 1.0, The Robert Gordon University, Aberdeen, UK).

### Human sample processing and analysis

The fasted blood samples were collected directly into heparinised tubes at days 0 and 7 of each intervention diet. The samples were centrifuged (1500 × g, 15 min; 4 °C) within 45 min to separate the plasma. The 24 h urine collection was measured by weight and volume at days 0 and 7 of each intervention diet. The harvested plasma and urine were aliquoted and stored at − 80 °C. The faecal samples were also collected at days 0 and 7 of each intervention diet and 5 g of faeces were mixed with 10 mL PBS buffer containing 30% glycerol before freezing, a 450 µL was aliquoted in Lysing Matrix E tubes of the FastDNa spin kit for soil (MB Biomedicals) and stored at -80 °C for DNA extraction [36]. The rest of the sample was immediately frozen at − 80 °C. Faecal samples were thawed, mixed once again, weighed and the faecal waters separated using a high-speed centrifuge (50,000 × g; 2 h; 10 °C) and then stored at − 80 °C prior LC-MS analysis. The deconjugation of metabolites in the urine and plasma samples was performed as previously described [24] and together with the faecal water samples were analysed using targeted LC-MS analysis as previously described [37]. The plasma, urine and faecal samples were analysed for metabolites predominantly produced by the phenylpropanoid pathway and products of protein and carbohydrate metabolism. These included derivatives and metabolites of the simple phenols, benzoic acids, phenolic acids, phenylacetic acids, phenypropionic acids, phenylpyruvic acids, phenyllactic acids, mandellic acids, phenolic dimmers, acetophenones, benzaldehydes, cinnamaldehydes, benzyl alcohols, cinnamyl alcohols, isoflavones, coumarins, chalcones, flavanones, flavones and flavonols. Heterocyclic amines, nitrosamines, indoles, polyamines, other protein metabolites and bile acids were also measured. The faecal water samples were also analysed for short-chain fatty acids (SCFA) as previously described [34].

The plasma metabolite lipid profiles (cholesterol, high-density lipoprotein; low-density lipoprotein; triglycerides, non-esterified fatty acids), glucose, insulin, urea and homocysteine (HCys) were measured as previously described [37–39].

The bacterial DNA extractions were performed with a FastDNA spin kit for soil (M.P. Biomedicals, Illkirch, France) according to the manufacturer’s instructions. DNA concentrations were measured using Qubit 2·0 Fluorometer (Thermo Fisher Scientific, Wilmington, DE, USA). The total number of 16 S rRNA gene copies per mL and abundance of several bacterial genera or species of the communities in the anaerobic faecal incubation experiments was determined by quantitative PCR as described previously [36] with 2 ng DNA in a total volume of 10 µL and expressed as 16 S rRNA gene copies per ml of culture. For the quantification of *Faecalibacterium prausnitzii*, primers used are described previously [41, 28] and for the quantification of bacteria related to the *Roseburia* genus primers used were Rrec2 described previously as well [42].

### Statistical analysis

Based on previous research [34], the recruitment of 16 participants per intervention would provide 90% power for detection of changes (> 45%) in the production of certain metabolites in plasma, urine and faecal samples. Therefore, this study will have > 90% power for detection with 20 participants (each volunteer will receive both study interventions in a randomised cross-over design) when the metabolites of macronutrients and phytochemicals are measured in the plasma, urine and faecal samples. Study had more than a single primary outcome studying diet differences found across a wide variety of metabolites in plasma, urine and faecal samples. Considering a typical coefficient of variation in these metabolites was about 50% for many metabolites of potential interest; a CV = 0.5 and D = 0.45 then used for the sample size calculation for differences in paired values, with alpha = 0.05 and power = 0.9 to obtain *n* = 16, recruiting 20, allowing for potential drop out. Furthermore, a power calculation for hunger scores was also conducted; a sample size of 20 subjects has also ∼90% power to detect differences comparable to within-subject variability (Cohen’s d = 1.0). Computer-generated random numbers were used to assign the subjects in pairs to first receive either the fava bean or buckwheat diet.

All data was reported as mean ± standard deviation (SD). To determine whether there were significant changes in plasma, faecal and urine metabolites between the habitual and intervention diets, paired t-Tests, equivalent to one sample tests comparing the mean change to zero, were used. Differences between diets were also assessed by paired t-Tests of the differences in each diet between baseline and endpoint. Microsoft Excel was used for all calculations, processing, and analysis of data in this study.

All metabolites data was analysed using principal component analysis (PCA), unit variance (U.V.)-scaled using SIMCA 14.1 (Umetrics, Cambridge).

## Results

### Volunteers characteristics

Twenty healthy participants (14 females, 6 males) with a mean age of 42.1 ± 12.1 years and BMI of 24.7 ± 4.7 kg/m^2^ were recruited to the study.

### Composition of the intervention diets

#### Nutrient composition

Proximate content of the buckwheat and fava bean intervention diets (as average of *n* = 4 days of four different days of buckwheat and respectively four different days of fava bean from the four-day rotation menu of 2000 kcal/day) is shown in Table 1A. The two intervention diets on average were not different in the macronutrient content apart from the total non-starch polysaccharides (NSP), which were significantly higher on average across the fava bean diets (*p* = 0.03). For the fava bean diets, the soluble NSP ranged between 1.2 and 1.61 g/day, while the insoluble NSP ranged between 24.5 and 38.04 g/day. For the buckwheat-based diets, the soluble NSP content ranged between 0.81 and 1.82 g/day, and the insoluble NSP was between 14.35 and 20.73 g/day (supplementary **Table S2)**. The monosaccharide composition of the soluble and insoluble NSP for each of the four days of the two diets is shown in supplementary **Table S2.** The WinDiets Nutritional Analysis found significant differences in the fat and carbohydrate content (%) between the participants’ habitual diet and the buckwheat-based diet (recorded using a seven-day weighed food diary) (supplementary **Figure S1**).

**Table 1:** Proximate values, the dry matter, ash, fat, total carbohydrate, resistant starch, crude protein and total non-starch polysaccharides (NSP) of the buckwheat and Fava bean diets in grams per day (for a representative 2000 kcal of 4 days rotation menu diet as part of the seven days intervention diet) and diet averages of *n* = 4 days **±** SD (**A**). The amino acid composition (excluding tryptophan, non-quantified) of the buckwheat and Fava bean intervention diets in grams per day (for a representative 2000 kcal diet) (**B**). The values are averages of *n* = 4 days diet **±** SD as part of the seven days intervention diet for histidine (His), Serine (Ser), arginine (Arg), glycine (Gly), aspartic acid (Asp), glutamic acid (Glu), threonine (Thr), Alanine (Ala), proline (Pro), lysine (Lys), tyrosine (Tyr), valine (Val), isoleucine (ILeu), leucine (Leu), phenylalanine (Phe), methionine (Met) and cysteine (Cys). Main micronutrient minerals expressed in mg ± sd per day (*n* = 4 day as part of the seven days intervention diet, for a representative 2000 kcal diet) measured in Fava bean and buckwheat diets (**C**). Differences between diets were also assessed by paired t-Tests, with p value less than 0.05 being significant

Proximate values	Buckwheat	Fava bean	*P* value (Buckwheat vs. Fava bean)
	Average (g/day ± SD)		
Dry matter	434.7 ± 41.2	492.4 ± 73.0	0.350
Ash	18.2 ± 1.2	19.2 ± 3.3	0.560
Total Fat	53.3 ± 5.4	67.0 ± 7.7	0.100
Total Carbohydrate	206.7 ± 30.3	207.1 ± 44.6	0.990
Resistant Starch	2.7 ± 1.0	4.5 ± 1.1	0.140
Crude Protein	72.9 ± 8.5	80.7 ± 17.2	0.560
Total NSP	18.9 ± 3.3	33.5 ± 5.5	0.030
**Essential** amino acids	Buckwheat	Fava bean	P value (Buckwheat vs. Fava bean)
	Average (g/day ± SD)		
His	2.0 ± 0.4	1.8 ± 0.2	0.250
Ileu	3.7 ± 0.6	3.1 ± 0.3	0.120
Leu	7.1 ± 1.5	5.8 ± 0.4	0.110
Lys	5.3 ± 0.9	5.2 ± 0.4	0.440
Met	0.9 ± 0.3	1.3 ± 0.2	0.110
Phe	3.9 ± 0.9	3.4 ± 0.3	0.180
Thr	3.5 ± 0.5	3.2 ± 0.4	0.280
Val	4.9 ± 0.9	4.4 ± 0.4	0.240
Trp	nq	nq	
**Non-essential** amino acids	Buckwheat	Fava bean	P value (Buckwheat vs. Fava bean)
	Average (g/day ± SD)		
Ser	5.2 ± 0.9	4.7 ± 0.6	0.290
Arg	6.0 ± 1.2	5.9 ± 0.9	0.470
Gly	3.3 ± 0.7	3.3 ± 0.4	0.500
Asp	10.5 ± 1.9	9.1 ± 0.8	0.200
Glu	22.2 ± 6.5	15.7 ± 1.2	0.070
Ala	4.1 ± 0.6	3.5 ± 0.5	0.190
Pro	6.5 ± 1.7	4.4 ± 0.2	0.040
Tyr	3.0 ± 0.8	2.5 ± 0.1	0.150
Cys	0.8 ± 0.4	0.8 ± 0.16	0.440
**Main** micronutrient minerals	Buckwheat	Fava bean	P value (Buckwheat vs. Fava bean)
	Average (mg/day ± SD)		
Sodium	1757.7 ± 230.0	2604.3 ± 685.2	0.029
Magnesium	588.0 ± 107.2	310.6 ± 48.7	0.002
Phosphorus	1601.8 ± 332.9	1182.4 ± 150.2	0.031
Potassium	2634.9 ± 167.3	3073.7 ± 388.0	0.042
Calcium	758.7 ± 87.2	800.2 ± 183.3	0.348
Chromium	0.06 ± 0.02	0.13 ± 0.09	0.083
Selenium	0.06 ± 0.02	0.05 ± 0.02	0.262
Molybdenum	0.09 ± 0.01	0.25 ± 0.61	0.001
Manganese	4.51 ± 0.78	4.47 ± 0.79	0.471
Iron	11.5 ± 2.6	15.2 ± 2.9	0.054
Copper	1.8 ± 0.3	2.0 ± 0.4	0.173
Zinc	9.6 ± 1.4	9.4 ± 0.65	0.397

The amino acid content of the buckwheat and fava bean diets (g/day, *n* = 4 ± SD) is shown in Table 1B as an average of the four-day rotation menus (2000 kcal/day) for the buckwheat and fava bean diets. Both intervention diets met the daily recommended nutrient intake (RNI) requirements for amino acids [43], except for cysteine. The overall amino acid composition of the intervention diets was comparable, except for proline, which was significantly higher in the buckwheat-rich diet.

The mineral content of the fava bean and buckwheat diets are presented in Table 1C. The fava-bean diet provided the participants with the daily RNI [44] requirements for most micronutrient minerals except potassium (RNI: 3500 mg/day, fava bean diet-based provided on average 3073.7 mg) and selenium (RNI: 0.075 mg/day, fava bean-based diet provided on average 0.05 mg). The fava bean-based diet had significantly higher sodium, potassium, iron and molybdenum levels compared to the buckwheat-based diet, but significantly less magnesium, phosphorus and selenium (Table 1C). Additionally, the buckwheat-based diet did not provide RNI levels for iron (RNI: 14.8 mg/day, buckwheat-based diet provided on average 11.5 mg), molybdenum (RNI: 0.225 mg/day, buckwheat-based diet provided on average 0.09 mg) and selenium levels (RNI: 0.075 mg/day, buckwheat-based diet provided on average 0.06 mg.

#### Phytochemicals composition

The most abundant phytochemicals measured in the fava bean and buckwheat-based diets are presented in supplementary **Figure S2**. The PCA plot of all the phytochemicals measured by LC-MS showed a clear separation between all the fava bean and buckwheat diets, demonstrating different profiles of the two intervention diets. Indole-3-pyruvic acid was the most abundant metabolite in both diets and approximately twice as abundant in the fava bean diet compared to the buckwheat diet. Ferulic acid was the second most abundant metabolite measured in the fava bean diet and epicatechin in the buckwheat diet.

### Satiety and hunger

There were no significant changes in subjectively rated satiety or hunger scores for either intervention diet compared to the volunteers’ habitual diets or when comparing the fava bean with buckwheat diets. Both intervention diets were able to successfully deliver similar appetite control compared to the volunteers’ habitual diets. Similarly, no notable differences were measured between the fava bean and buckwheat diets regarding satiety or hunger. Small differences were observed in the quantity of food consumed between the habitual and intervention diets, with the intervention diets providing slightly lower quantities of foods (see online supplementary **Figures S3A-D**). The Windiet nutritional composition analysis of the volunteers’ habitual diets vs. the intervention diets (see online supplementary **Figures S4A-G)** revealed that the volunteers consumed less calories, fat, protein and carbohydrates (during the fava bean diet only) and higher amounts of fibre during both intervention diets.

### Fasted plasma homocysteine, insulin, urea, glucose and lipids profiles

There was a significant diet effect on fasted plasma homocysteine following the consumption of the intervention diets (Day 0 vs. Day 7 of the diets), with the fava bean diet significantly increasing (*p* = 0.022) and the buckwheat diet significantly decreasing (*p* = 0.0018) homocysteine (Table 2). Both diets significantly decreased plasma urea, and the fava bean diet significantly reduced fasted plasma glucose and insulin following seven days of consumption. Furthermore, both diets significantly increased plasma non-esterified fatty acids (NEFA) values.

**Table 2:**
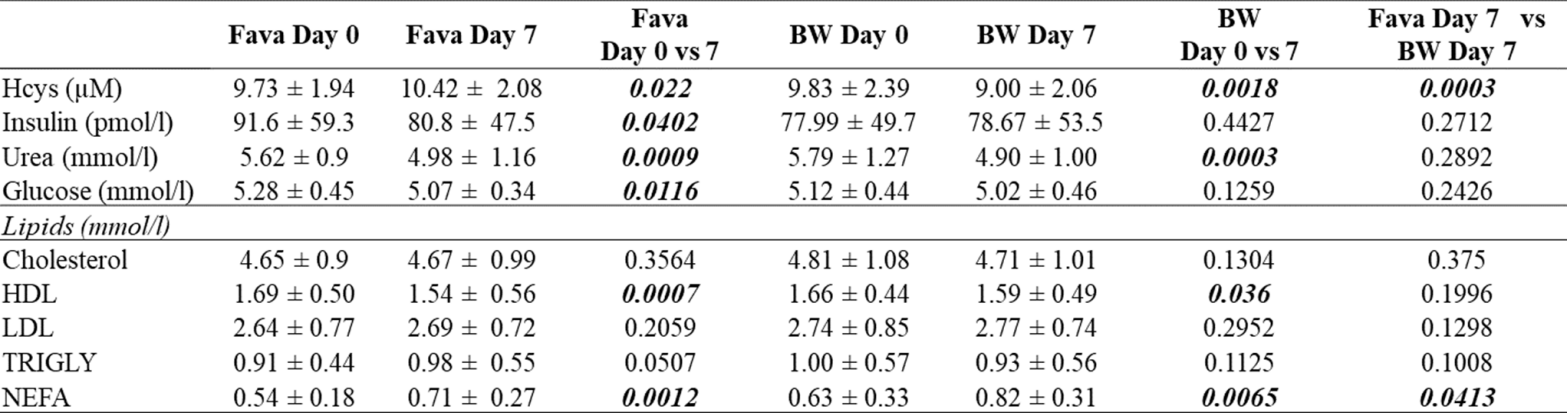
The average values of volunteers (*n* = 20) ± SD fasted plasma homocysteine, insulin, urea, glucose and lipids (cholesterol HDL, LDL, triglycerides, NEFA) at day 0 and day 7, prior to and after consumption of Fava bean and buckwheat intervention diets, respectively. The p values measured using tTest for day 0 vs. day 7 of each diet and day 7 vs. day 7

BW: buckwheat; Fava: Fava bean; *Hcys = homocysteine* HDL: high-density lipoprotein; LDL: low-density lipoprotein; TRIGLY: triglycerides, NEFA: non-esterified fatty acids

### Additional metabolites measured in plasma, urine and faecal samples

**Fig. 2:**
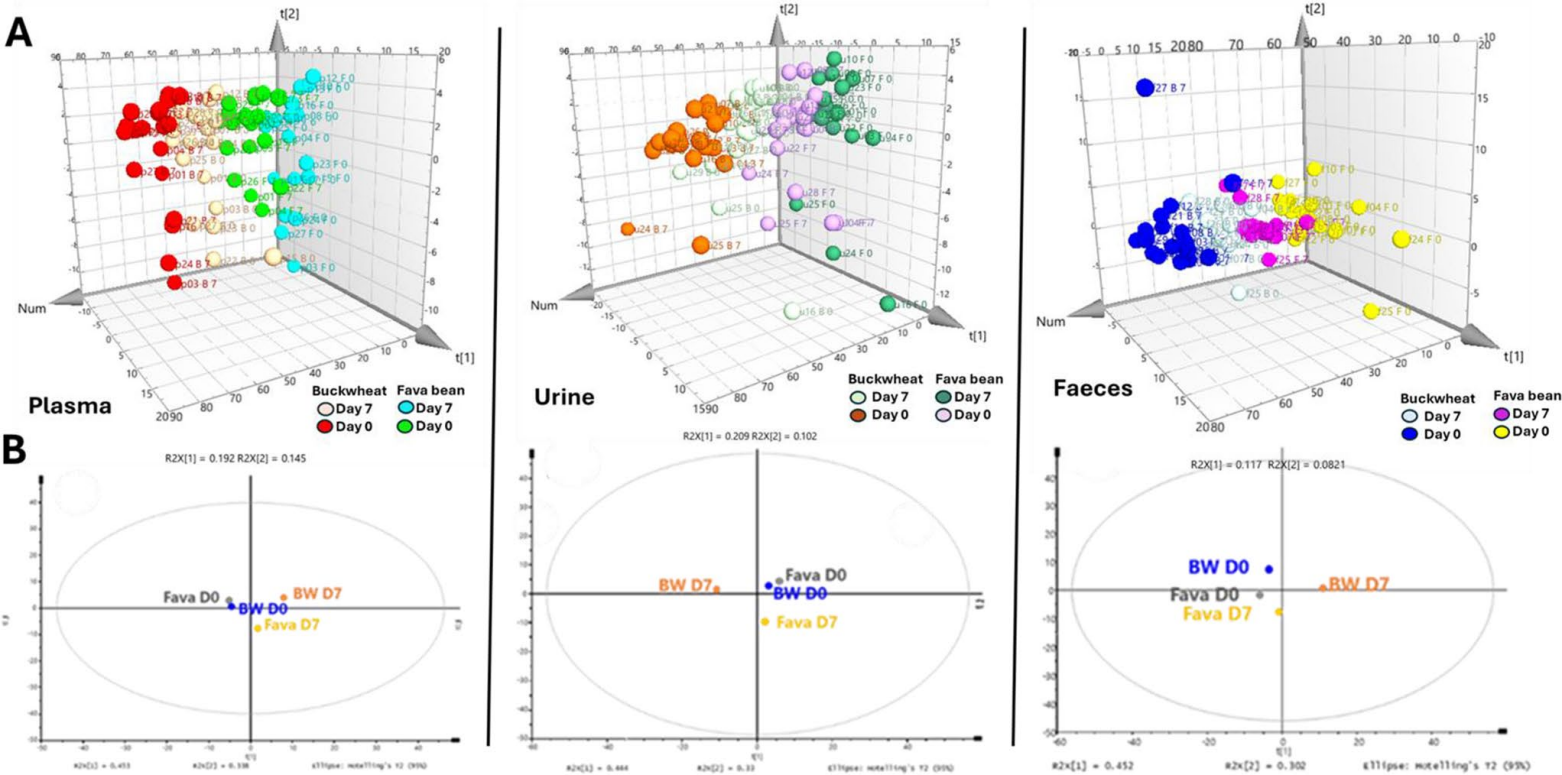
PCA scatter 3D plot analysis of LC-MS metabolites profiles measured in individual volunteers’ plasma, urine and faeces at Day 0 and Day 7 before and after fava bean and buckwheat diet consumption (**A**). PCA analysis (average profile *n* = 20 volunteers) of plasma, urine and faecal metabolites before (Day 0) and after (Day 7) fava bean (Fava D0, grey; Fava D7 yellow) and buckwheat (BW D0 blue, BW D7 orange) diet consumption (**B**)

One hundred and eighty metabolites were analysed in volunteers’ plasma, urine and faecal samples using targeted LC-MS analysis at Day 0 and Day 7 of each intervention diet. Specifically, metabolites produced by the phenylpropanoid pathway and products of protein and carbohydrate metabolism as specified in material and methods section. Figure 2A shows the PCA analysis of these metabolites profile in each volunteer’ biological fluids and the Fig. 2B shows the PCA for the metabolites profile for all the volunteers in average at Day 0 and 7 of each diet. The average plasma and urine metabolites profiles at Day 0 for both intervention diets are similar, as they are grouped in the same quadrant whereas, their profiles differ at Days 7, as they are in different quadrants from Day 0 and from each diet. In the case of the faecal metabolites (Fig. 2B), buckwheat diet has a more pronounced effect on the faecal metabolome as the Day 0 and Day 7 distributions are in different quadrants while the metabolites at Day 0 and Day 7 for the fava bean diet are in the same quadrant.

Out of 180 metabolites analysed, only four metabolites were significantly changed in the plasma samples, 17 in the urine samples and 24 in the faecal samples following the consumption of the fava bean diet (Day 0 vs. Day 7) (see Table 3). Following the consumption of the buckwheat diet 15 metabolites were significantly changed in the plasma samples, 108 in the urine samples and ten in the faecal samples (Day 0 vs. Day 7) (Table 3).

**Table 3:** Average concentration (pg/µL, *n* = 20 ± SD) of plasma metabolites at day 0 (baseline) and day 7 with a significant change following consumption of Fava bean diet and buckwheat diet for 7 days. Average concentration (*n* = 20 ± SD) of urine metabolites (measured in pg/µL) on day 0 (baseline) and day 7 with a significant change following consumption of the Fava bean diet and buckwheat-based diet for 7 days. Average concentration (*n* = 20 ± SD) of faecal metabolites (measured in pg/µL) on day 0 (baseline) and day 7 with a significant change following consumption of the Fava bean diet and buckwheat-based diet for 7 days. BW: buckwheat; Fava: Fava bean

	Plasma Samples (Average ± SD, pg/µL)		
	Fava Day 0	Fava Day 7	Day 0 vs. 7
			(*p*-value)
vanillic acid	1168.8 ± 160.7	1261.9 ± 155.4	0.028
piperidine	2211.6 ± 202.4	2319.6 ± 196.8	0.034
3,4-dimethoxycinnamic acid	19.5 ± 2.4	21.1 ± 4.3	0.038
tangeretin	13.5 ± 12.6	10.7 ± 9.4	0.045
	**BW Day 0**	**BW Day 7**	**Day 0 vs. 7**
			**(** ***p*** **-value)**
salicylic acid	188.2 ± 24.8	738.9 ± 268.2	< 0.001
quinadilic acid	19.5 ± 7.1	44.2 ± 19.5	< 0.001
*o*-hydroxyhippuric acid	18.6 ± 11.1	215.6 ± 87.5	< 0.001
2,3-dihydroxybenzoic acid	8.4 ± 21.4	30.5 ± 20.1	0.002
phenylpropionic acid	47.9 ± 117.0	196.1 ± 208.2	0.002
p-hydroxybenzoic acid	3479.0 ± 196.4	3641.0 ± 247.9	0.010
glycoursodeoxycholic acid	95.0 ± 118.3	46.1 ± 51.1	0.009
4-hydroxy-3-methoxymandelic acid	123.6 ± 13.9	137.9 ± 18.1	0.016
2,6-dihydroxybenzoic acid	42.8 ± 15.4	34.9 ± 7.6	0.016
putrescine	20901.0 ± 1019.6	21530.0 ± 1253.7	0.027
enterolactone	22.2 ± 23.5	38.9 ± 20.5	0.027
glycochenodeoxycholic acid	796.7 ± 757.2	445.6 ± 250.6	0.031
indole-3-propionic acid	484.7 ± 413.2	324.1 ± 153.5	0.039
protocatechualdehyde	0.8 ± 1.9	3.4 ± 5.1	0.041
4-hydroxyphenylacetic acid	2049.6 ± 193.4	2160.4 ± 118.7	0.041
**Urine Samples (Average ± SD**,** pg/µL)**			
	**Fava Day 0**	**Fava Day 7**	**Day 0 vs. 7** **(** ***p-*** **value)**
3,5-dihydroxybenzoic acid	306.7 ± 303.6	1089.0 ± 813.4	0.001
indole-3-lactic acid	980.3 ± 310.6	852.2 ± 283.9	0.001
indole-3-carbinol	3.3 ± 14.9	52.1 ± 66.0	0.004
indole-3-propionic acid	42.2 ± 46.1	85.3 ± 94.3	0.004
anthranilic acid	465.9 ± 500.7	254.7 ± 245.2	0.008
3,4,5-trimethoxycinnamic acid	30.2 ± 48.7	6.6 ± 15.8	0.013
psoralen	7.8 ± 3.2	4.7 ± 3.2	0.015
4-methoxyphenylacetic acid	247.2 ± 113.7	322.5 ± 168.4	0.019
epicatechin	7.1 ± 10.6	2.5 ± 6.8	0.019
p-hydroxybenzaldehyde	186.7 ± 57.8	152.3 ± 47.4	0.022
8-methylpsoralen	38.8 ± 5.0	36.7 ± 4.0	0.023
2,6-dihydroxybenzoic acid	163.5 ± 99.5	117.0 ± 42.8	0.024
4-hydroxyphenylacetic acid	5619.9 ± 3295.2	4036.0 ± 1515.4	0.028
syringic acid	244.6 ± 133.8	174.9 ± 65.9	0.034
enterolactone	1904.7 ± 1541.6	1162.5 ± 549.1	0.037
p-hydroxybenzoic acid	2733.1 ± 700.3	2343.4 ± 802.3	0.042
hydroxytyrosol	9.9 ± 9.8	18.7 ± 15.2	0.045
	**BW Day 0**	**BW Day 7**	**Day 0 vs. 7** **(** ***p –*** **value)**
salicylic acid	190.6 ± 76.9	1151.6 ± 391.0	< 0.001
o-hydroxyhippuric acid	1346.4 ± 1156.2	14936.0 ± 6763.4	< 0.001
salicyl alcohol	0.0 ± 0.0	263.6 ± 119.1	< 0.001
2,3-dihydroxybenzoic acid	57.8 ± 46.1	656.7 ± 405.8	< 0.001
p-hydroxybenzoic acid	2585.7 ± 503.2	4320.1 ± 1337.7	< 0.001
quinadilic acid	580.1 ± 366.9	1276.3 ± 673.6	< 0.001
4-methoxyphenylacetic acid	309.1 ± 121.7	712.8 ± 364.5	< 0.001
3-hydroxyphenylacetic acid	1532.9 ± 846.0	3326.7 ± 1768.7	< 0.001
2,5-dihydroxybenzoic acid	270.6 ± 267.6	1109.0 ± 951.8	0.001
3,4,5-trimethoxycinnamic acid	45.1 ± 79.5	164.2 ± 206.1	0.001
catechin	13.2 ± 15.4	44.4 ± 38.8	0.002
enterlactone	1895.2 ± 1824.7	4112.5 ± 2005.6	0.003
daidzein	250.1 ± 264.0	54.2 ± 28.1	0.003
indole-3-propionic acid	46.0 ± 46.9	20.4 ± 39.2	0.005
4-hydroxy-3-methoxyphenylpropionic acid	1548.8 ± 822.8	1002.5 ± 229.8	0.007
3,5-dihydroxybenzoic acid	300.4 ± 330.7	54.4 ± 243.3	0.008
2,6-dihydroxybenzoic acid	179.6 ± 95.7	122.8 ± 55.2	0.011
glycitein	175.2 ± 180.7	63.8 ± 11.5	0.012
genstein	71.1 ± 58.6	37.6 ± 8.3	0.014
vanillin	21.4 ± 13.9	11.6 ± 11.7	0.021
scopoletin	60.0 ± 27.8	43.7 ± 9.6	0.022
ellagic acid	95.6 ± 179.8	731.7 ± 1326.9	0.043
3,4-dimethoxybenzoic acid	99.6 ± 101.4	140.1 ± 81.2	0.047
**Fecal water Samples (Average ± SD**,** pg/µL)**			
	**Fava Day 0**	**Fava Day 7**	**Day 0 vs. 7** **(** ***p-*** **value)**
luteolinidin	4.9 ± 7.7	11.4 ± 8.8	0.001
p-coumaric acid	30.6 ± 46.9	11.6 ± 29.1	0.002
caffeic acid	78.9 ± 76.2	20.6 ± 26.3	0.002
deoxycholic acid	13648.8 ± 11659.6	10277.8 ± 9128.2	0.004
2-amino-3,8-dimethylimidazo[4,5-f] quinoxaline	1.9 ± 3.4	5.3 ± 5.5	0.011
protocatachaldehyde	19.3 ± 16.8	6.02 ± 12.6	0.012
coniferyl alcohol	47.8 ± 49.4	18.5 ± 37.9	0.012
protocatechuic acid	100.8 ± 93.1	53.7 ± 45.0	0.013
ellagic acid	108.6 ± 175.4	0 ± 0	0.014
enterloctone	599.9 ± 616.1	219.4 ± 84.4	0.019
Fisetin	161.3 ± 144.2	120.3 ± 94.9	0.020
enterodiol	114.8 ± 142.8	28.7 ± 16.8	0.020
2-amino-1-methyl-6-phenylimidazo[4,5-b] pyridine	1.9 ± 2.3	1.1 ± 1.9	0.026
syringic acid	81.7 ± 133.0	6.3 ± 15.0	0.026
phloretin	0.4 ± 0.6	0.02 ± 0.1	0.028
myricetin	114.8 ± 124.8	63.5 ± 74.8	0.031
secoisolariciresinol	39.5 ± 72.8	0.5 ± 2.1	0.031
quercetin	5.3 ± 7.3	1.6 ± 3.2	0.035
vanillic acid	53.2 ± 76.4	11.0 ± 23.9	0.037
tyromine	157.5 ± 207.4	69.0 ± 74.2	0.041
2,6-dihydroxybenzoic acid	7.6 ± 11.5	1.7 ± 7.2	0.046
phenylacetic acid	86434.2 ± 32741.6	72411.1 ± 35524.4	0.046
syringin	4.9 ± 8.1	0.6 ± 2.6	0.047
chenodeoxycholic acid	546.0 ± 964.3	114.0 ± 260.9	0.047
	**BW Day 0**	**BW Day 7**	**Day 0 vs. 7** **(** ***p –*** **value)**
m-anisic acid	1.8 ± 7.5	102.7 ± 84.8	< 0.001
5-hydroxy tryptophan	132.4 ± 238.2	457.4 ± 400.9	< 0.001
2-amino-3,4-dimethylimidazo[4,5-f] quinoxaline	1.15 ± 3.2	8.9 ± 4.9	< 0.001
2-amino-3,4,8-trimethylimidazo[4,5-f] quinoxaline	6.1 ± 1.2	4.2 ± 0.6	< 0.001
hydrogenated ferulic dimer H5-5	6747.4 ± 5846.4	1558.7 ± 1949.0	< 0.001
tyrosol	217.1 ± 93.8	128.2 ± 71.2	< 0.001
ferulic dimer (5–5 linked)	34.2 ± 39.8	7.2 ± 18.1	< 0.001
ferulic acid	75.4 ± 69.9	15.3 ± 25.8	0.005
Fisetin	165.3 ± 161.7	58.1 ± 64.8	0.005
2,3-dihydroxybenzoic acid	28.4 ± 17.9	282.6 ± 340.2	0.007
p-anisic acid	0 ± 0	23.9 ± 35.3	0.007
spermidine	3829.1 ± 4333.4	2090.3 ± 1939.3	0.009
4-hydroxy-3-methoxyphenylpropionic acid	503.6 ± 348.7	227.2 ± 244.7	0.011
daidzein	8.2 ± 11.4	0.7 ± 2.9	0.012
spermine	133.9 ± 98.7	77.3 ± 20.0	0.013
quercetin	3.6 ± 4.6	8.2 ± 6.3	0.013
2-amino-3,8-dimethylimidazo[4,5-f] quinoxaline	3.8 ± 5.6	0.8 ± 2.01	0.013
3,4,5-trimethoxybenzaldehyde	0 ± 0	0.8 ± 1.4	0.016
3,5-dihydroxybenzoic acid	44.1 ± 69.2	5.2 ± 16.2	0.020
salicylic acid	115.3 ± 95.9	661.2 ± 1004.4	0.020
2-Amino-1-methyl-6-phenylimidazo(4,5-b)pyridine	2.4 ± 2.6	1.3 ± 2.4	0.020
2,5-dihydroxybenzoic acid	143.8 ± 171.4	456.6 ± 568.8	0.024
3,4-dimethoxybenzoic acid	121.03 ± 196.3	76.5 ± 145.5	0.024
syringic acid	93.0 ± 112.6	22.9 ± 28.4	0.030
indole-3-propionic acid	641.4 ± 414.3	474.1 ± 324.2	0.033
gallic acid	23.8 ± 36.6	122.3 ± 179.3	0.038
3,4-dihydroxyphenylpropionic acid	629.6 ± 434.6	395.8 ± 275.6	0.040
p-hydroxybenzoic acid	116.2 ± 104.8	274.3 ± 327.8	0.042
deoxycholic acid	16635.6 ± 14305.8	12070.3 ± 10490.7	0.047
apigenin	1.1 ± 2.0	0.05 ± 0.22	0.048

There were only eight metabolites significantly different in plasma samples following the consumption for seven days of the fava bean diet compared to seven days of buckwheat diet (Day 7 vs. Day 7). Apart from indole 3-propionic acid, which was significantly higher at 7 days following the fava bean diet, all the other metabolites were significantly higher at Day 7 following consumption of the buckwheat diet. However, the indole 3-propionic acid was not significantly increased following the fava diet when compared with baseline (0 vs. 7 days), (Table 4). A total of 32 metabolites were found to be significantly different in the volunteers’ urine samples following consumption for seven days of the fava bean diet compared to the buckwheat diet. More than half (19) of these metabolites were at significantly higher concentrations after consuming the buckwheat diet, (Day 7 vs. Day 7, Table 4). Thirty-nine faecal metabolites showed a significant difference, ten being significantly higher following the fava bean and 20 following the buckwheat diet in faeces collected at day seven following both diets (Day 7 vs. Day 7, Table 4).

**Table 4:** Plasma, urine, and faecal metabolites on day 7 following Fava and buckwheat-based diets

Plasma samples	Fava Day 7	BW Day 7	Day 7 vs. 7 (*p*-value)
	Average ± SD, pg/µL plasma		
0-hydroxyhippuric acid	20.0 ± 23.2	215.6 ± 87.5	< 0.001
salicylic acid	206.7 ± 106.0	738.9 ± 268.2	< 0.001
enterlactone	10.9 ± 5.6	38.9 ± 20.5	< 0.001
quinadilic acid	16.7 ± 9.9	44.2 ± 19.5	< 0.001
2,3-dihydroxybenzoic acid	3 ± 9.2	30.5 ± 20.1	< 0.001
phenylpropionic acid	14.8 ± 66.2	196.1 ± 208.2	< 0.001
indole-3-propionic acid	569.6 ± 328.0	324.1 ± 153.5	0.002
anthranilic acid	13.7 ± 4.5	16.9 ± 3.5	0.002
**Urine samples**	**Fava Day 7**	**BW Day 7**	**Day 7 vs. 7** **(** ***p–*** **value)**
	**Average ± SD**,** pg/µL urine**		
salicylic acid	193.6 ± 72.8	1151.6 ± 391.0	< 0.001
o-hydroxyhippuric acid	976.8 ± 570.1	14936.0 ± 6763.4	< 0.001
p-hydroxybenzoic acid	2343.4 ± 802.3	4320.1 ± 1337.7	< 0.001
2-hydroxy benzyl alcohol	2.3 ± 10.4	263.6 ± 119.1	< 0.001
enterolactone	1162.5 ± 549.1	4112.5 ± 2005.6	< 0.001
2,3-dihydroxybenzoic acid	61.4 ± 54.0	656.7 ± 405.8	< 0.001
quinadilic acid	458.1 ± 308.5	1276.3 ± 673.6	< 0.001
3,5-dihydroxybenzoic acid	1089.0 ± 813.4	54.4 ± 243.3	< 0.001
4-methoxyphenylacetic acid	322.5 ± 168.4	712.8 ± 364.5	< 0.001
hydroxytyrosol	18.7 ± 15.2	3.2 ± 3.0	< 0.001
catechin	3.2 ± 8.2	44.4 ± 38.8	< 0.001
3-hydroxyphenylacetic acid	1612.8 ± 857.6	3326.7 ± 1768.7	< 0.001
3,4-dimethoxybenzoic acid	77.4 ± 77.6	140.1 ± 81.2	< 0.001
2,5-dihydroxybenzoic acid	344.2 ± 269.9	1109.0 ± 951.8	0.001
3,4,5-trimethoxycinnamic acid	18.7 ± 15.2	164.2 ± 206.1	0.002
daidzein	315.2 ± 335.1	54.2 ± 28.1	0.002
indole-3-acrylic acid	38.1 ± 30.9	15.2 ± 10.1	0.003
p-cresol	91.1 ± 50.8	168.0 ± 104.9	0.003
indole-3-propionic acid	85.3 ± 94.3	20.4 ± 39.2	0.005
scopoletin	54.7 ± 16.9	43.7 ± 9.6	0.007
p-hydroxybenzaldehyde	152.3 ± 47.4	199.6 ± 67.3	0.008
genstein	60.9 ± 35.0	37.6 ± 8.3	0.009
glycitein	106.3 ± 65.5	63.8 ± 11.5	0.011
epicatechin	2.5 ± 6.8	26.6 ± 43.7	0.013
bergapten	11.3 ± 5.0	14.5 ± 4.9	0.016
ferulic acid	6340.5 ± 3954.6	4081.2 ± 2408.8	0.016
4-hydroxyphenylacetic acid	4036.0 ± 1515.4	5581.0 ± 2979.6	0.017
isoliquiritigenin	0.8 ± 0.3	0.7 ± 0.2	0.021
hydrogenated ferulic dimer h5-5	57.8 ± 14.6	50.4 ± 10.3	0.027
ellagic acid	70.2 ± 104.5	731.7 ± 1326.9	0.031
enterodiol	109.8 ± 106.2	856.4 ± 1533.4	0.035
4-hydroxy-3-methoxyphenylpropionic acid	1331.8 ± 664.9	1002.5 ± 229.8	0.045
**Faecal samples**	**Fava Day 7**	**Buckwheat Day 7**	**Day 7 vs. 7** **(** ***p –*** **value)**
	**Average ± SD**,** pg/µL faecal water**		
2-amino-3,4-dimethylimidazo[4,5-f] quinoxaline	0.6 ± 2	8.9 ± 4.9	< 0.001
2-amino-3,4,8-trimethylimidazo[4,5-f] quinoxaline	5.3 ± 0.8	4.2 ± 0.6	< 0.001
enterlactone	219.4 ± 84.4	1102.9 ± 645.7	< 0.001
m-anisic acid	0 ± 0	102.7 ± 84.8	0.0001
luteolinidin	11.4 ± 8.8	0.3 ± 1.4	0.0001
reservatrol	1 ± 2.5	18 ± 15	0.0001
fisetin	120.3 ± 94.9	58.1 ± 64.8	0.0003
1,2-hydroxybenzene	66.3 ± 83.4	153.6 ± 122.9	0.0003
apigenin	1.7 ± 1.5	0.1 ± 0.2	0.0003
phenylpropionic acid	20051.9 ± 9627.6	30020.3 ± 11074.9	0.0004
3,5-dihydroxybenzoic acid	62.1 ± 56	5.2 ± 16.2	0.0005
quercetin	1.6 ± 3.2	8.2 ± 6.3	0.0007
hydrogenated Ferulic Dimer H5-5	4425.1 ± 3936.4	1558.7 ± 1949	0.0009
2-amino-3,8-dimethylimidazo[4,5-f] quinoxaline	5.3 ± 5.5	0.8 ± 2	0.0012
tyrosol	211.6 ± 104.1	128.2 ± 71.2	0.0018
spermine	115.6 ± 58.8	77.3 ± 20	0.0040
quinadilic acid	24.1 ± 18	104 ± 98.9	0.0040
2,3-dihydroxybenzoic acid	28 ± 21.2	282.6 ± 340.2	0.0049
2,5-dihydroxybenzoic acid	139.5 ± 143.1	456.6 ± 568.8	0.0063
p-anisic acid	0 ± 0	23.9 ± 35.3	0.0082
putresine	6009.5 ± 7894	5034.7 ± 7355.5	0.0112
indole-3-propionic acid	811 ± 721.7	474.1 ± 324.2	0.0116
5-OHtryptophan	205.1 ± 354	457.4 ± 400.9	0.0128
gallic acid	11.5 ± 21.8	122.3 ± 179.3	0.0131
p-hydroxybenzoic acid	91 ± 127	274.3 ± 327.8	0.0138
salicylic acid	59.4 ± 69.6	661.2 ± 1004.4	0.0141
protocatachaldehyde	6 ± 12.6	19 ± 18.8	0.0152
phenylacetic acid	72411.1 ± 35524.4	96144.7 ± 30529.5	0.0160
3,4,5-trimethoxybenzaldehyde	0 ± 0	0.8 ± 1.4	0.0172
ellagic acid	0 ± 0	284.7 ± 421.3	0.0174
spermidine	4874.4 ± 6345.9	2090.3 ± 1939.3	0.0189
ferulic dimer (5–5 linked)	28.7 ± 34	7.2 ± 18.1	0.0201
syringic acid	6.3 ± 15	22.9 ± 28.4	0.0207
enterodiol	28.7 ± 16.8	281.3 ± 406.8	0.0294
morin	73.8 ± 69.5	47.5 ± 43.5	0.0358
daidzein	5.3 ± 8.3	0.7 ± 2.9	0.0368
3-hydroxyphenylacetic acid	2524.7 ± 1617.6	5192.1 ± 6287.2	0.0381
coniferyl alcohol	18.5 ± 37.9	54.3 ± 75.1	0.0381
4-hydroxy-3-methoxyphenylpropionic acid	621.5 ± 701.7	227.2 ± 244.7	0.0493

BW: buckwheat; Fava: Fava bean

Consumption of the buckwheat and fava-bean diets for seven days did not increase SCFA production concentrations in faecal samples. The levels of individual SCFAs (acetic, propionic, and butyric acids) and total SCFA concentrations in faecal samples was not significantly different following both interventions **(**supplementary **Figures S5 and S6).**

### Gut microbiota composition following the consumption of the intervention diets

The response of the microbiota to the buckwheat and fava bean diets was investigated by quantitative PCR (Table 5**)**. Total bacteria as well as 22 specific microbial groups covering the dominant members of the microbiota, were enumerated in 17 volunteers for whom a full set (Day 0 and Day 7) of faecal samples was available. Statistical analysis revealed significant changes in some of the bacterial groups, five significant changes following the buckwheat diet and two after fava bean dietary intervention. Specifically, consumption of buckwheat diet significantly decreased the *Roseburia* group, *Anaerostipes hadrus* species, *Bifidobacterium adolescentis*, *Dorea* spp. and *Bifidobacterium* spp. (compared with baseline Day 0 vs. Day 7, Table 5). Following the fava bean diet intervention, only *Lactobacillus* species decreased significantly (*p* < 0.05), whereas *Coprococcus eutactus* increased significantly (*p* < 0.05). *(A) hadrus* (*p* < 0.05), *Bifidobacterium* species (*p* < 0.01) and *(B) adolescentis* (*p* < 0.05) were significantly higher at Day 7 after fava bean in comparison with Day 7 buckwheat diet (Day 7 vs. Day 7, Table 5).

**Table 5:** Abundance of total faecal microbiota and specific genera or species (average 16 S rRNA gene copies/g faeces ± sem, *n* = 17) during habitual (baseline) diet and day 7 (D7) of the intervention diet consumption, determined by qPCR. ANOVA with terms for volunteer, baseline and diet was used to compare bean hull with control. qPCR, quantitative PCR data analysed 0 vs. 7 and 7 vs. 7 days of intervention diets using paired t test analysis, where * *p* < 0.05, ** *p* < 0.01

	Fava baseline	Fava D7	BW baseline	BW D7
		16 S copies/g faeces		
Total bacteria	6.48E + 10 ± 1.57E + 10	6.61E + 10 ± 1.60E + 10	9.03E + 10 ± 2.19E + 10	7.03E + 10 ± 1.15E + 10
*Bacteroides* spp.	5.71E + 09 ± 1.39E + 09	7.69E + 09 ± 1.86E + 09	8.18E + 09 ± 1.98E + 09	7.08E + 09 ± 1.97E + 09
*Prevotella* spp.	2.12E + 09 ± 5.13E + 08	2.08E + 09 ± 5.04E + 08	2.91E + 09 ± 7.06E + 08	9.28E + 08 ± 3.40E + 08
Ruminococcaceae	1.18E + 10 ± 2.87E + 09	1.32E + 10 ± 3.21E + 09	1.73E + 10 ± 4.20E + 09	1.50E + 10 ± 3.00E + 09
*Faecalibacterium prausnitzii*	4.20E + 09 ± 1.02E + 09	5.23E + 09 ± 1.27E + 09	6.32E + 09 ± 1.53E + 09	5.36E + 09 ± 1.12E + 09
*Ruminococcus bromii*	2.01E + 09 ± 4.88E + 08	2.40E + 09 ± 5.81E + 08	2.67E + 09 ± 6.47E + 08	2.92E + 09 ± 5.89E + 08
*Ruminococcus albus* group	4.66E + 08 ± 1.13E + 08	8.98E + 08 ± 2.18E + 08	7.87E + 08 ± 1.91E + 08	1.18E + 09 ± 7.42E + 08
*Ruminococcus flavefaciens* group	1.11E + 09 ± 2.69E + 08	1.36E + 09 ± 3.30E + 08	1.05E + 09 ± 2.54E + 08	1.49E + 09 ± 3.39E + 08
*Oscscillibacter* group	2.12E + 09 ± 5.14E + 08	1.78E + 09 ± 4.32E + 08	3.48E + 09 ± 8.43E + 08	2.24E + 09 ± 4.64E + 08
Negativicutes	6.58E + 08 ± 1.60E + 08	6.36E + 08 ± 1.54E + 08	9.25E + 08 ± 2.24E + 08	7.23E + 08 ± 1.70E + 08
*Roseburia* group	2.84E + 09 ± 6.88E + 08	2.68E + 09 ± 6.50E + 08	**5.20E + 09 ± 1.26E + 09** ^¥**^	**2.02E + 09 ± 5.00E + 08** ^¥**^
*Anaerobutyricum hallii* ^a^	7.76E + 08 ± 1.88E + 08	7.91E + 08 ± 1.92E + 08	8.78E + 08 ± 2.13E + 08	8.14E + 08 ± 2.00E + 08
*Anaerostipes hadrus*	4.50E + 08 ± 1.09E + 08	**5.86E + 08 ± 1.42E + 08** ^§*^	**5.56E + 08 ± 1.35E + 08** ^¥*^	**3.41E + 08 ± 7.12E + 07** ^§¥*^
*Coprococcus eutactus*	**5.28E + 08 ± 1.28E + 08** ^¥*^	**7.73E + 08 ± 1.87E + 08** ^¥*^	6.37E + 08 ± 1.55E + 08	4.28E + 08 ± 1.55E + 08
*Dorea* spp.	9.84E + 08 ± 2.39E + 08	8.07E + 08 ± 1.96E + 08	**1.52E + 09 ± 3.68E + 08** ^¥**^	**7.43E + 08 ± 1.47E + 08** ^¥**^
*Lachnospira eligens* ^b^	5.11E + 08 ± 1.24E + 08	6.54E + 08 ± 1.59E + 08	3.54E + 08 ± 8.60E + 07	5.23E + 08 ± 1.34E + 08
*Blautia* spp.	3.67E + 09 ± 8.91E + 08	3.48E + 09 ± 8.45E + 08	4.78E + 09 ± 1.16E + 09	3.89E + 09 ± 9.37E + 08
*Lactobacillus* spp.	**1.36E + 07 ± 3.29E + 06** ^¥*^	**4.93E + 06 ± 1.20E + 06** ^¥*^	1.45E + 07 ± 3.51E + 06	1.36E + 07 ± 6.80E + 06
*Bifidobacterium* spp.	1.68E + 09 ± 4.06E + 08	**1.41E + 09 ± 3.43E + 08** ^§**^	**2.05E + 09 ± 4.97E + 08** ^¥*^	**5.73E + 08 ± 1.70E + 08** ^§**¥*^
*Bifidobacterium adolescentis*	7.15E + 08 ± 1.73E + 08	**5.89E + 08 ± 1.43E + 08** ^§*^	**6.41E + 08 ± 1.55E + 08** ^¥*^	**2.11E + 08 ± 8.40E + 07** ^§*¥*^
*Desulfovibrio* spp.	5.43E + 08 ± 1.32E + 08	5.54E + 08 ± 1.34E + 08	6.90E + 08 ± 1.67E + 08	6.00E + 08 ± 2.23E + 08
Enterobbacteria	1.06E + 08 ± 2.58E + 07	2.02E + 08 ± 4.89E + 07	2.19E + 08 ± 5.31E + 07	5.65E + 07 ± 1.95E + 07
Methanogens	3.46E + 08 ± 8.39E + 07	3.46E + 08 ± 8.39E + 07	2.58E + 08 ± 6.26E + 07	2.85E + 08 ± 8.16E + 07

a This genus has been renamed from Eubacterium to Anaerobutyricum

b This genus has been renamed from Eubacterium to Lachnospira

¥ Significant differences between Day 0 and Day 7 following an intervention diet

§Significant differences at Day 7 between the two intervention diets

BW: buckwheat; Fava: fava bean

## Discussion

### The Fava bean diet was superior in iron and trace elements while the buckwheat diet excels in magnesium and selenium

Both the fava bean and buckwheat-based diets supplied essential micronutrients that met or exceeded the Recommended Nutrient Intake (RNI) for sodium, magnesium (300 mg/day), phosphorus (625 mg/day), calcium (700 mg/day), manganese (2.3 mg/day), copper (1.2 mg/day), zinc (9.5 mg/day), and chromium (0.025 mg/day). However, neither diet achieved the RNI for selenium and potassium. The buckwheat diet further fell short in iron and molybdenum. Fava bean provided significantly greater levels of potassium, sodium, chromium, iron, and molybdenum, whereas buckwheat offered more magnesium, phosphorus, and selenium.

Given that iron and zinc deficiencies are prominent contributors to micronutrient malnutrition or “hidden hunger”, a condition independent of caloric deficiency and associated with energy-dense, nutrient-poor diets [45], the higher iron content in fava bean and zinc content in both diets positions these plant sources as valuable strategies for combatting such deficiencies. Their integration into regular dietary patterns could support more sustainable approaches to meet mineral intake requirements and address widespread malnutrition. Therefore, in vivo mineral bioavailability from buckwheat and fava bean should be assessed.

### Complementing plant-based protein sources with animal sources offset lower DIAAS of plant protein

Both intervention diets provided all indispensable amino acids except cysteine, with only minor differences observed, specifically in proline content—between fava bean and buckwheat [9, 46]. The Digestible Indispensable Amino Acid Score (DIAAS), which has replaced the Protein Digestibility-Corrected Amino Acid Score (PDCAAS) as the standard for protein quality, places both fava bean (59–61%) [47] and buckwheat (54–63%) [48] below the threshold for a quality claim (< 75%).

However, the inclusion of complementary protein sources—eggs, potatoes, and cheese—within the dietary menus likely enhanced the composite DIAAS, illustrating that combining plant-based foods with select animal protein can effectively compensate for lower individual protein quality. This approach underscores the potential for dietary biodiversity to support optimal amino acid provision while fostering sustainability.

### Both study diets sustain satiety despite lower energy and macronutrient consumption compared with volunteers’ habitual diets

Satiety, hunger, and desire to eat, measured via visual analogue scales (VAS) from 07:00 to 23:00, showed no significant differences between the fava bean and buckwheat diets or compared to habitual intake. Despite delivering fewer calories due to reduced intake of fat, protein, and carbohydrates (particularly in the fava bean group), both intervention diets led to greater fibre consumption and maintained perceived satiety. The fava bean diet exceeded daily fibre requirements with 33.5 g of non-starch polysaccharides (NSP; 112% RNI), while the buckwheat diet supplied 18.9 g NSP (63% RNI). The habitual diet provided only 76% of recommended fibre intake [49]. These findings highlight the capacity of dietary fibre consumption to potentially compensate for maintaining the satiety levels, as there were no differences in the hunger between habitual and intervention diets contributing to these feelings of fullness. Although faecal SCFA concentrations did not increase, colonic absorption may explain the unchanged levels. Enhancing the fermentability of fava bean fibre could promote greater SCFA production. Similar observations were done in a previous study where high fibre breads were reformulated using fava bean hull flour and consumed by healthy volunteers several days [50].

### The Fava bean diet lowers fasting glucose and insulin; and the buckwheat diet reduces homocysteine

After one week, the fava bean diet significantly reduced fasting glucose and insulin levels, whereas the buckwheat diet showed only a trend toward reduced glucose. These effects are likely attributable to higher fibre intake in the fava bean group, in line with evidence linking dietary fibre to improved insulin sensitivity and glucose control [51]. This is an important finding, since this effect was observed after only one week of dietary intervention. Longer-term intake of fava bean foods should be investigated for the impact on glucose metabolism to see if there is value in promoting this crop as part of nutritional strategy to prevent and/or manage T2D.

Both diets also lowered serum urea, an indicator of renal and cardiovascular disease risk [52]. Interestingly, homocysteine concentrations increased significantly after the fava bean diet but decreased markedly following buckwheat consumption. Although elevated homocysteine is associated with cardiovascular and neurodegenerative diseases [52–54], vitamin supplementation strategies targeting homocysteine have failed to yield expected clinical outcomes [55–58], suggesting it serves as a risk marker rather than a direct cause. A diet rich in buckwheat could therefore potentially counteract factors that increase heart disease risk, such as a diet high in red meat and low in fruits and vegetables or smoking.

### The buckwheat diet boosts anti-inflammatory and antioxidant compounds, and the Fava bean diet enhances β-cell protective metabolites

Several metabolites linked to potential health benefits were positively influenced by the buckwheat diet. Notably, salicylic acid and 2,3-dihydroxybenzoic acid levels rose significantly across all biological fluids post-consumption. Salicylic acid, a plant metabolite mainly produced via the phenylpropanoid pathway, is known for its anti-inflammatory effects [60]. It may reduce oxidative stress-related T2D complications by slowing glucose absorption [61, 62] and has been shown to improve glucose tolerance and lower fasting glucose levels in both diabetic and non-diabetic individuals [62, 63]. The microbial flavonoid metabolite 2,3-dihydroxybenzoic acid has been shown to regulate glucose uptake and production in renal NRK-52E cells [64]. O-hydroxyhippuric acid levels also increased significantly in plasma and urine after buckwheat intake. Hippuric acid, a microbial metabolite, is positively associated with uric acid excretion [65] and negatively linked to visceral fat [66], blood pressure [67], and metabolic syndrome [68]. Additionally, glycochenodeoxycholic acid decreased significantly in fasted plasma after buckwheat consumption, a beneficial effect given its role in hepatocyte apoptosis and necrosis [69]. Other health-related metabolites like 5-hydroxytryptophan, gallic acid, and p-hydroxybenzoic acid also increased significantly in faecal samples following buckwheat diet.

The consumption of fava bean diet significantly increased urinary levels of indole-3-propionic acid (IPA) and 3,5-dihydroxybenzoic acid. Diets rich in fibre and whole grains promote IPA synthesis [70] and 3,5-dihydroxybenzoic acid formation [71]. IPA is produced from tryptophan via Stickland fermentation in the gut [72] and may protect beta-cell function, lowering the risk of T2D in glucose-intolerant individuals [73, 74]. 3,5-dihydroxybenzoic acid could support liver detoxification, reduce inflammation, stimulate intestinal cell renewal, and promote blood cell formation [71]. Additionally, deoxycholic acid, a secondary bile acid linked to bowel tumorigenesis via mucosal and DNA damage [75], was significantly reduced in faecal samples after fava (and buckwheat) consumption. These findings highlight fava beans and buckwheat as promising ingredients for development of functional foods for promoting systemic health and guiding nutritional therapies.

### The fava bean diet preserves beneficial taxa while buckwheat diet alters microbial diversity

Gut microbiota composition differed significantly between baseline and intervention phases of both diets. The buckwheat diet reduced the abundance of several health-associated bacterial taxa, including the butyrate-*producing Roseburia genus*,* Anaerostipes hadrus* [76], and *Bifidobacterium* species [77]. These reductions were not seen with the fava bean diet, except for a significant decrease in Lactobacillus species.

Notably, one week of fava bean consumption significantly increased *Coprococcus eutactus*, the only bacterium to show a significant rise. This is the first human study linking fava bean intake to increased *C. eutactus*. This species produces butyrate and is among the 15 gut microbes reduced in individuals with Parkinson’s disease [77–79]. *C. eutactus* is also depleted in children with delayed language development and adults with Parkinson’s disease [81]. As fava beans naturally contain levodopa, a key treatment for Parkinson’s, their consumption may offer additional benefits for those with the condition [81–83]. Further randomized controlled trials are needed to explore the relationship between fava bean intake, gut microbiota, and mental health.

Post-intervention, *A. hadrus* and *Bifidobacterium* species were significantly more abundant following the fava bean diet than the buckwheat diet, possibly due to its higher fibre content. Overall, microbiota shifts were modest, consistent with the lack of change in faecal SCFA concentrations and total bacterial load. Longer dietary interventions may be required for more pronounced effects.

## Study limitations

The present study has several limitations. As the study describes how fava bean and buckwheat, rich diets could sustainably diversify and help the nutrient requirements, it would have been useful compare these results with that of a meat-rich diet. However, this study compared both diets (nutrient composition and intake) with the volunteers’ habitual diets which also contained meat, which successfully tested the study aims. Furthermore, the study has not measured the impact of the intervention diets on plasma and urine concentrations of B6 and B12 vitamins, nor studied the in vivo mineral bioavailability from the fava bean and buckwheat foods. Another limitation is that in this study WinDiets Nutritional Analysis was used to compare both the habitual diets and intervention diets composition. Wet chemistry methodologies were used to analyse the nutrient composition of representative 2000 kcal intervention diets (as explained prior) and we couldn’t use wet chemistry methodology to analyse the habitual and all other intervention diets. However, using both WinDiets and wet chemistry to analyse the intervention diets, independently of the analysis used there were significant differences between the amount of dietary fibre consumed in the intervention diets analysed.

## Conclusions

Buckwheat and fava beans are nutrient-dense crops that support dietary diversification while contributing to more sustainable and resilient food systems. In this study, diets enriched with buckwheat and fava bean were found to be equally satiating compared to participants’ habitual diets, while also beneficially modulating a range of health-associated metabolites. These metabolic shifts are particularly relevant to the prevention of cardiovascular disease and type 2 diabetes. Notably, this study is the first to demonstrate that a fava bean–rich diet significantly increases the abundance of *Coprococcus eutactus*, a butyrate-producing gut bacterium associated with neurological and metabolic health. These findings underscore the potential of buckwheat and fava bean as functional food ingredients in strategies aimed at improving cardiometabolic and gut health.

## Electronic supplementary material

Below is the link to the electronic supplementary material.

Supplementary Material 1

Supplementary Material 2
