# Supplementary material for: Fava bean and buckwheat are sustainable food sources which support satiety and beneficially modulate several biomarkers, bacteria and metabolites associated with human health
Source: Eur J Nutr. 2025 Jun 7;64(5):211. doi: 10.1007/s00394-025-03726-6 (PMC12145301; doi:10.1007/s00394-025-03726-6)
Supplement: Supplementary file 2 — Supplementary Material 2 [file 394_2025_3726_MOESM2_ESM.docx]

Supplementary Files


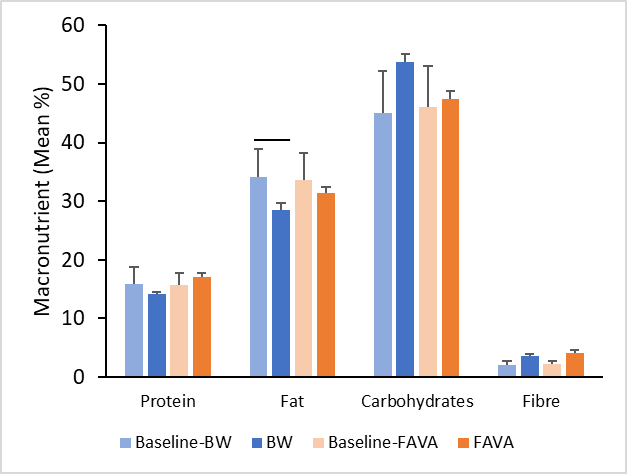


*

*

**Supplementary Figure S1:** Mean % of the habitual and intervention diets Macronutrient composition between fava-based diet and buckwheat-based diet. There were significant differences in fat and carbohydrate levels for the buckwheat-based diet


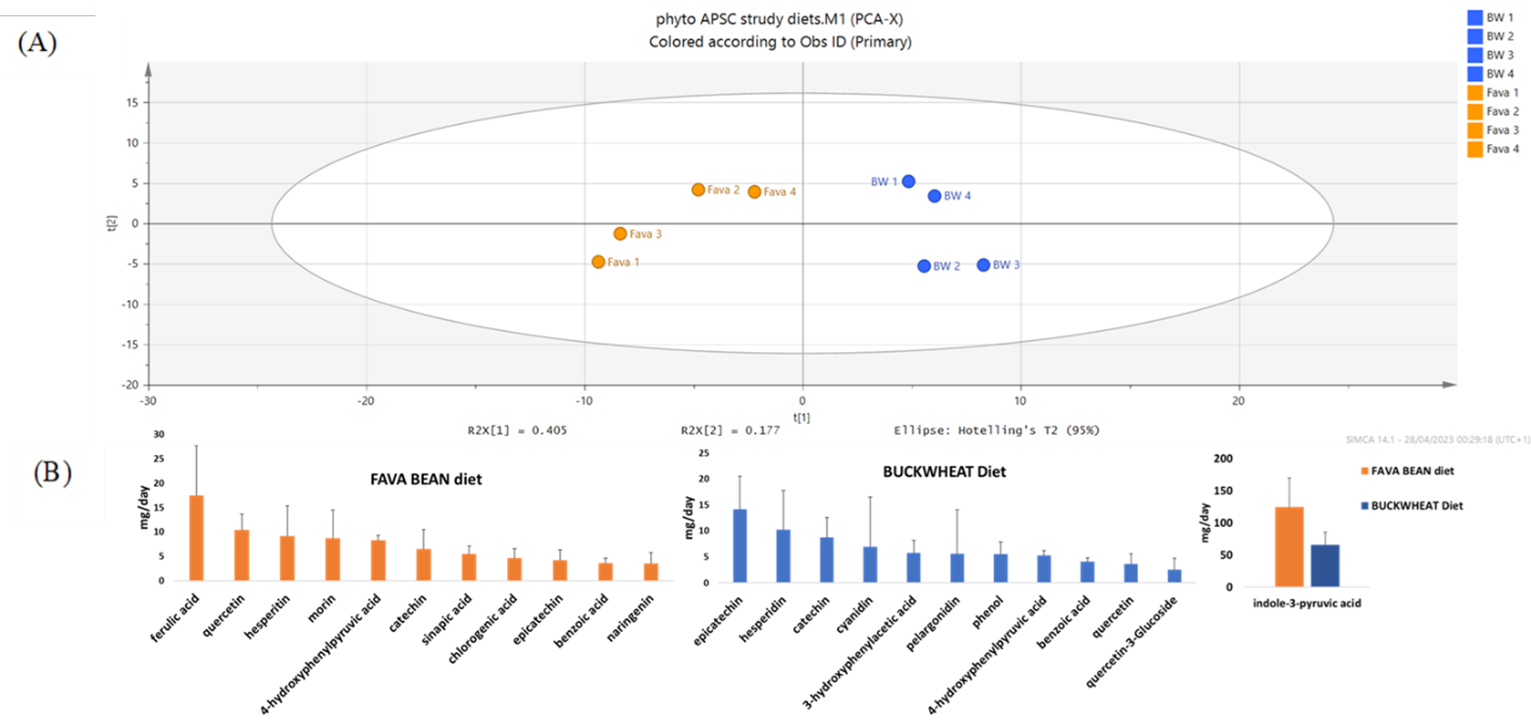


Supplementary **Figure S2.** Plant metabolites measured in fava bean (Fava) and buckwheat (BW)-based diets: (A) Principal Component Analysis (PCA) (scaled) of diet metabolites from four days of fava bean (orange) and buckwheat diets (based on daily consumption of 2000kcal) (B) Most abundant metabolites with average concentrations (n=4 days diet ± SD) over 5 mg per day measured in the fava and respectively buckwheat diets

1. **Baseline Buckwheat vs Baseline Fava bean-based diets**


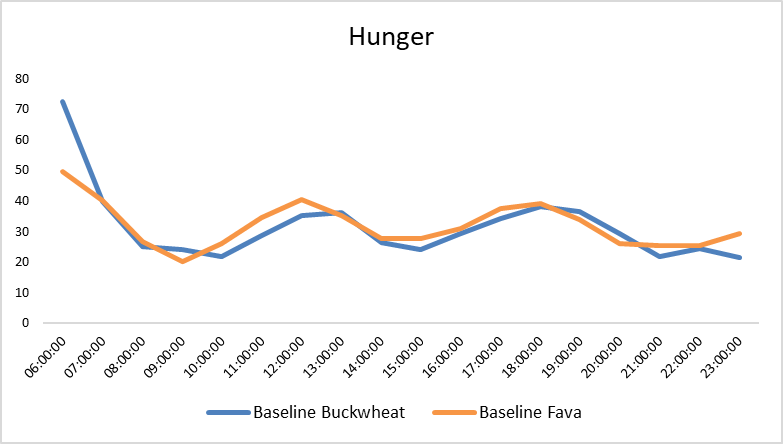


1. **Baseline Buckwheat based diet and postintervention buckwheat**
2. **Baseline Fava bean based diet and postintervention Fava bean based diet**

1. **Buckwheat based and fava bean based diets**

**Supplementary Figure** S3 (A) Hunger, fullness, quantity desire and appetite average scores for the habitual (baseline) diets prior the intervention diets (buckwheat and fava diets) of twenty volunteers during the wake up hours (6am-11pm) of the day for one week. (B and C) Hunger, fullness, quantity, desire and appetite average scores in habitual (baseline) vs during each intervention diet (buckwheat (B) and fava bean(C) of twenty volunteers during the wake up hours (6am-11pm) of the day for one week. (D) Hunger, fullness, quantity, desire and appetite average scores during the intervention diets fava bean vs buckwheat of twenty volunteers during the wake up hours (6am-11pm) of the day for one week.

**Supplementary Figure S4 –** Macronutrient composition of the habitual and intervention diets.

**Supplementary Figure 4 S5.** Microbial SCFA following the consumption of buckwheat based and fava bean diets for 7 days in 20 volunteers

**Supplementary Figure 5**.S6 Total SCFA concentrations following the consumption of buckwheat based and fava bean diets for 7 days in 20 volunteers.

**Table S2. Monosaccharide composition of soluble and insoluble NSP content in the buckwheat and fava-bean diets**

| **Diet (Day)** | **NSP** | **Rhamnose** | **Fucose** | **Arabinose** | **Xylose** | **Mannose** | **Galactose** | **Glucose** | **Uronic Acid** | **TOTAL NSP(%)** | **TOTAL NSP (SOL+INSOL)** | **TOTAL NSP per day (g)** | **NSP type per DAY (g)** |
| --- | --- | --- | --- | --- | --- | --- | --- | --- | --- | --- | --- | --- | --- |
| Fava 1 | Soluble | 0±0 | 0±0 | 0.02±0 | 0.03±0 | 0±0 | 0.01±0 | 0.01±0 | 0.15±0.02 | 0.23±0.02 | 5.37 | 33.71 | 1.43 |
|  | Insoluble | 0.04±0.01 | 0.03±0.01 | 0.6±0.02 | 0.95±0.02 | 0.15±0.01 | 0.27±0.02 | 2.31±0.08 | 0.8±0.07 | 5.14±0.22 |  |  | 32.27 |
|  |  |  |  |  |  |  |  |  |  |  |  |  |  |
| Fava 2 | Soluble | 0±0 | 0±0 | 0.01±0 | 0.02±0 | 0±0 | 0.01±0 | 0.02±0 | 0.28±0 | 0.34±0.01 | 7.33 | 34.94 | 1.6 |
|  | Insoluble | 0.06±0.01 | 0.03±0.01 | 0.67±0.02 | 0.8±0.03 | 0.09±0.01 | 0.39±0.02 | 2.83±0.15 | 2.12±0.12 | 7±0.34 |  |  | 33.34 |
|  |  |  |  |  |  |  |  |  |  |  |  |  |  |
| Fava 3 | Soluble | 0±0 | 0±0 | 0.03±0 | 0.05±0 | 0.01±0 | 0.01±0 | 0.02±0 | 0.21±0.01 | 0.33±0.01 | 5.39 | 26.09 | 1.61 |
|  | Insoluble | 0.03±0 | 0.03±0.01 | 0.46±0.02 | 0.78±0.03 | 0.18±0.01 | 0.28±0.02 | 2.56±0.09 | 0.74±0.03 | 5.05±0.19 |  |  | 24.48 |
|  |  |  |  |  |  |  |  |  |  |  |  |  |  |
| Fava 4 | Soluble | 0±0 | 0±0 | 0.01±0 | 0.02±0 | 0±0 | 0.01±0 | 0.01±0 | 0.22±0.01 | 0.27±0 | 8.49 | 39.26 | 1.23 |
|  | Insoluble | 0.06±0.01 | 0.04±0 | 0.99±0 | 1.68±0.04 | 0.19±0.01 | 0.33±0 | 3.03±0.07 | 1.91±0.03 | 8.23±0.04 |  |  | 38.04 |
|  |  |  |  |  |  |  |  |  |  |  |  |  |  |
| **Fava Average** | **Soluble** | **0** | **0** | **0.02** | **0.03** | **0** | **0.01** | **0.01** | **0.22** | **0.29** | **6.64** | **34.07** | **1.49** |
|  | **Insoluble** | **0.05** | **0.03** | **0.68** | **1.05** | **0.15** | **0.32** | **2.68** | **1.39** | **6.35** |  |  | **32.58** |
|  |  |  |  |  |  |  |  |  |  |  |  |  |  |
| Buckwheat 1 | Soluble | 0±0 | 0±0 | 0.01±0 | 0.01±0 | 0±0 | 0.02±0 | 0.01±0 | 0.14±0.01 | 0.2±0.01 | 3.89 | 15.92 | 0.81 |
|  | Insoluble | 0.05±0 | 0.04±0.01 | 0.32±0.01 | 0.58±0.02 | 0.18±0 | 0.27±0 | 1.46±0.05 | 0.81±0.03 | 3.69±0.06 |  |  | 15.11 |
|  |  |  |  |  |  |  |  |  |  |  |  |  |  |
| Buckwheat 2 | Soluble | 0±0 | 0±0 | 0.01±0 | 0.01±0 | 0±0.01 | 0.02±0 | 0.01±0 | 0.24±0.02 | 0.29±0.03 | 4.43 | 21.99 | 1.44 |
|  | Insoluble | 0.06±0.01 | 0.05±0 | 0.38±0.01 | 0.67±0.01 | 0.14±0.01 | 0.28±0 | 1.67±0.07 | 0.9±0.02 | 4.14±0.09 |  |  | 20.55 |
|  |  |  |  |  |  |  |  |  |  |  |  |  |  |
| Buckwheat 3 | Soluble | 0±0 | 0±0 | 0.01±0 | 0.01±0 | 0±0 | 0.02±0 | 0.02±0 | 0.13±0.02 | 0.18±0.03 | 4.52 | 21.6 | 0.87 |
|  | Insoluble | 0.05±0.01 | 0.04±0.01 | 0.37±0.03 | 0.31±0.04 | 0.15±0.02 | 0.33±0.02 | 2.26±0.08 | 0.83±0.08 | 4.34±0.2 |  |  | 20.73 |
|  |  |  |  |  |  |  |  |  |  |  |  |  |  |
| Buckwheat 4 | Soluble | 0±0 | 0±0 | 0.01±0.01 | 0.01±0.02 | 0±0 | 0.02±0 | 0.01±0.01 | 0.38±0.02 | 0.43±0.05 | 3.8 | 16.17 | 1.82 |
|  | Insoluble | 0.05±0 | 0.04±0.01 | 0.38±0.01 | 0.27±0.02 | 0.11±0 | 0.34±0 | 1.31±0.11 | 0.86±0.13 | 3.37±0.07 |  |  | 14.35 |
|  |  |  |  |  |  |  |  |  |  |  |  |  |  |
| **Buckwheat Average** | **Soluble** | **0** | **0** | **0.01** | **0.01** | **0** | **0.02** | **0.01** | **0.22** | **0.27** | **4.16** | **18.81** | **1.24** |
|  | **Insoluble** | **0.05** | **0.04** | **0.36** | **0.46** | **0.14** | **0.3** | **1.67** | **0.85** | **3.88** |  |  | **17.57** |
